# Supplementary material for: Dissociable neural mechanisms track evidence accumulation for selection of attention versus action
Source: Nat Commun. 2018 Jun 27;9:2485. doi: 10.1038/s41467-018-04841-1 (PMC6021379; doi:10.1038/s41467-018-04841-1)
Supplement: Supplementary file 1 — Supplementary Information [file 41467_2018_4841_MOESM1_ESM.pdf]

Dissociable neural mechanisms track evidence accumulation for selection of attention versus action

Amitai Shenhav, Mark A. Straccia, Sebastian Musslick, Jonathan D. Cohen, Matthew M. Botvinick

Supplementary Information

Supplementary Methods

Defining motion and color-sensitive regions
We included localizer scans at the end of each session to identify regions that were especially sensitive to dot motion (MT+) and color (V4). Following Kayser and colleagues[1], each localizer consisted of ten consecutive 40s blocks, with each block consisting of 10s of increased motion or color information followed by a 30s baseline. For the motion localizer, the 10s period consisted of 100% coherently moving dots, the direction of which changed every second (sampled randomly from the range 0°:36:324°**,** without replacement), and the 30s baseline consisted of static dots. The color localizer consisted of Mondrian-like images alternating every second in color (10s) versus grayscale (30s baseline).

MT+ and V4 ROIs were generated based on the motion and color localizer tasks, spatially constrained by a priori regions identified by Kayser and colleagues. We performed whole-brain analyses for both localizers, in each case examining regions whose activity was increased during the condition of interest (high-coherence motion or colorful Mondrians) relative to the relevant baseline (static dots or gray patches). The associated first-level analyses and contrasts proceeded as described in the main text. We then used each participant’s localizer to identify the 20 voxels within MT+ (6mm ROI centered at -46, -74, -2) most sensitive to motion and the 20 voxels within V4 (centered at -28, -84, -22) most sensitive to color.

Supplementary Figures

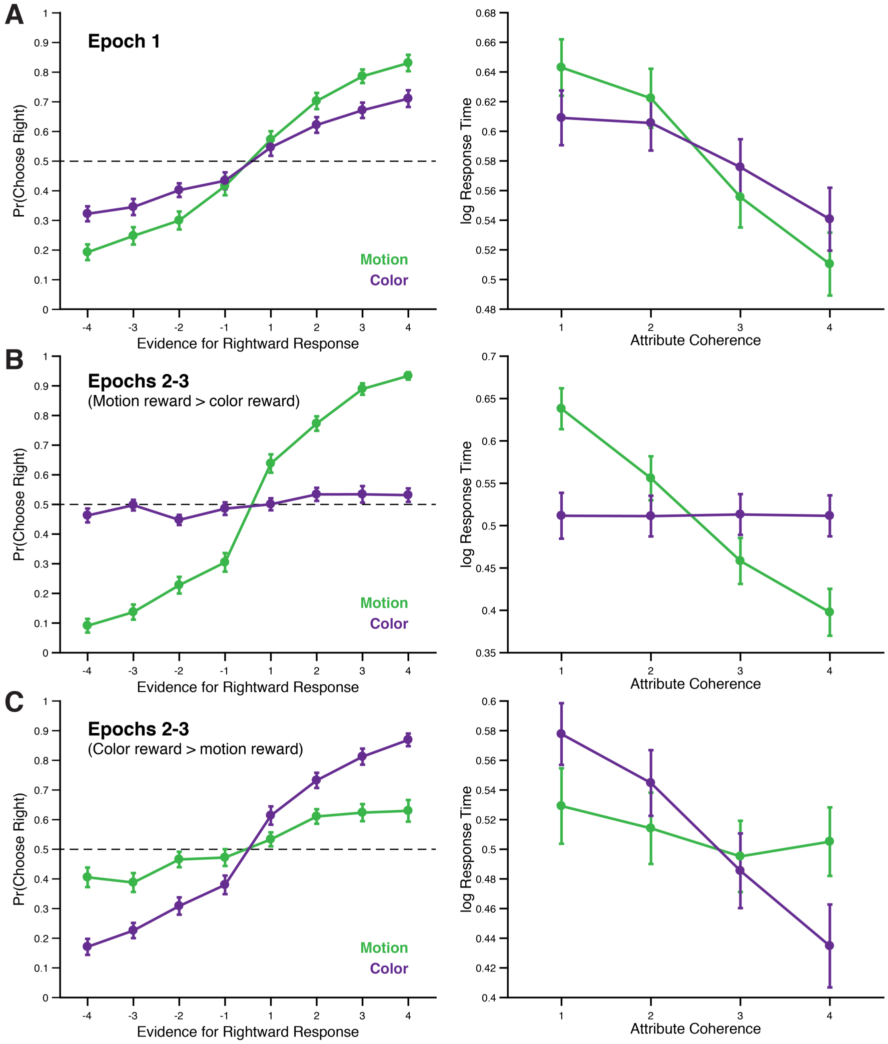

Supplementary Figure 1. Behavioral performance relative to motion and color evidence. **A)** During Epoch 1 (when color and motion were equally rewarded), choices and RTs were determined by the amount of evidence for both motion and color attributes, with a greater influence of motion evidence. **B-C)** During Epochs 2-3, behavior was primarily determined by the more rewarded attribute, whether that was motion (**B**) or color (**C**). Error bars reflect s.e.m.

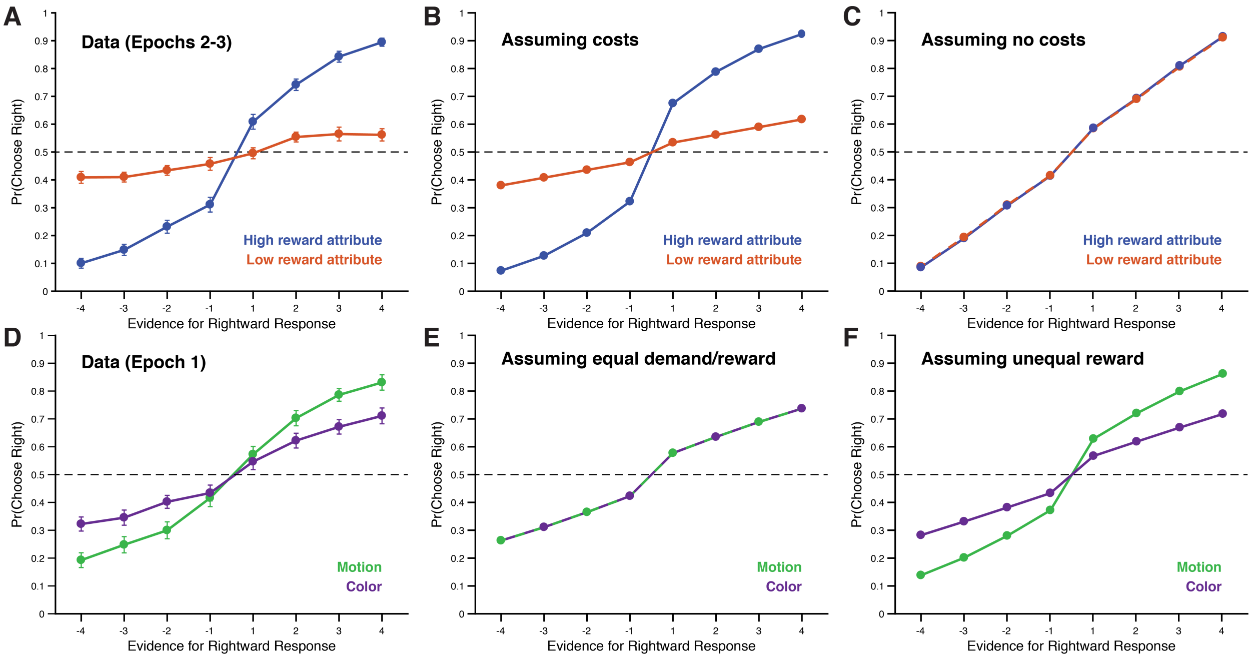

Supplementary Figure 2. Simulated performance based on value-maximizing allocation of attention. A-C) During the unequal-reward epochs, the EVC-maximizing allocation of attention (**B**) weights the high-reward attribute much more heavily than the low-reward attribute, similar to what we observe in our data (**A**) but unlike the normative allocation when attention is assumed not to have a cost (i.e., when it is only determined by reward rate; **C**), in which case attention is distributed across the two attributes equally. **D-F)** During Epoch 1, motion and color should be weighted equally if they are similarly demanding and if participants only consider extrinsic rewards (**E**). However, our observation that motion is weighted more heavily than color (**D**) can be obtained under several different conditions, including differences in intrinsic reward for the two attributes (**F**) as well as differences in discriminability or effort costs for the two attributes (not shown). While our experiment cannot distinguish between these possibilities, it is important to note that the rewards for these attributes were counterbalanced, avoiding potential confounds related to differences in attribute weighting.

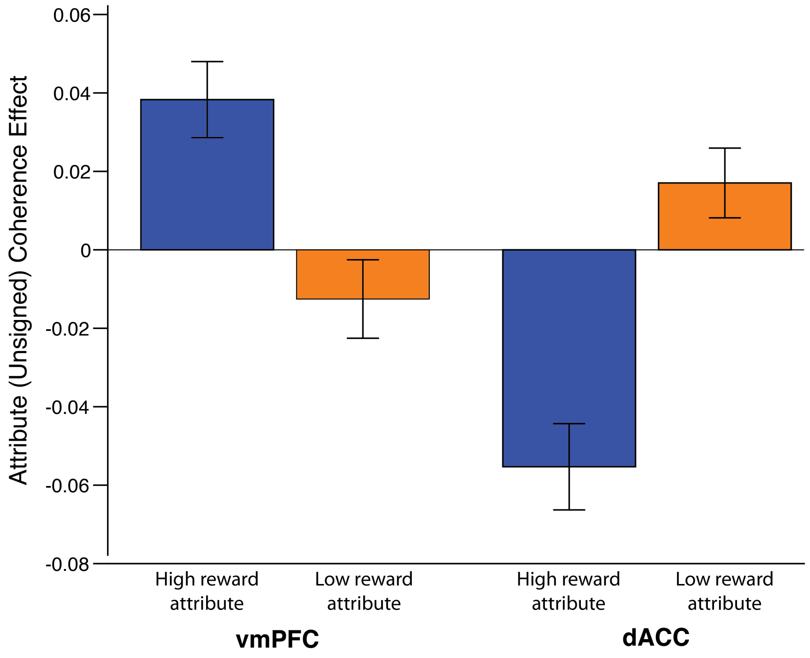

Supplementary Figure 3. vmPFC and dACC differentially encode the coherence of the high and low reward attributes. Whereas vmPFC positively tracked the (unsigned) coherence of the high-reward attribute and negatively tracked the coherence of the low-reward attribute, dACC exhibited the opposite pattern. Regression coefficients are plotted with corresponding s.e.m.

Supplementary References

1 Kayser, A. S., Erickson, D. T., Buchsbaum, B. R. & D'esposito, M. Neural representations of relevant and irrelevant features in perceptual decision making. *J. Neurosci.* **30**, 15778-15789, (2010).
